# A gel single ion conducting polymer electrolyte enables durable and safe lithium ion batteries *via* graft polymerization

**DOI:** 10.1039/c8ra07557c

**Published:** 2018-11-30

**Authors:** Yazhou Chen, Guodong Xu, Xupo Liu, Qiyun Pan, Yunfeng Zhang, Danli Zeng, Yubao Sun, Hanzhong Ke, Hansong Cheng

**Affiliations:** Sustainable Energy Laboratory, Faculty of Material Science and Chemistry, China University of Geosciences (Wuhan) 388 Lumo RD Wuhan 430074 China sunyubao@gmail.com kehanz@163.com +86 13377851282; School of Chemistry and Environmental Engineering, Yancheng Teachers University No. 2, Xiwang Avenue Yancheng 224007 Jiangsu Province China

## Abstract

Concentration polarization issues and lithium dendrite formation, which associate inherently with the commercial dual-ion electrolytes, restrict the performance of lithium ion batteries. Single ion conducting polymer electrolytes (SIPEs) with high lithium ion transference numbers (*t*_+_ ≈ 1) are being intensively studied to circumvent these issues. Herein, poly(ethylene-*co*-vinyl alcohol) (EVOH) is chosen as the backbone and then grafted with lithium 3-chloropropanesulfonyl(trifluoromethanesulfonyl)imide (LiCPSI) *via* Williamson's reaction, resulting in a side-chain-grafted single ion polymer conductor (EVOH-*graft*-LiCPSI). The ionomer is further blended with poly(vinylidene fluoride-*co*-hexafluoropropylene) (PVDF-HFP) by solution casting for practical use. The SIPE membrane with ethylene carbonate and dimethyl carbonate (EC/DMC = 1 : 1, v/v) as plasticizer (*i.e.*, gel SIPE) exhibits an ionic conductivity of 5.7 × 10^−5^ S cm^−1^, a lithium ion transference number of 0.88, a wide electrochemical window of 4.8 V (*vs.* Li/Li^+^) and adequate mechanical strength. Finally, the gel SIPE is applied in a lithium ion battery as the electrolyte as well as the separator, delivering an initial discharge capacity of 100 mA h g^−1^ at 1C which remains at 95 mA h g^−1^ after 500 cycles.

## Abstract 2

A post-grafting strategy to synthesize single ion conducting polymer electrolytes for lithium metal secondary batteries.

## Graphical abstract

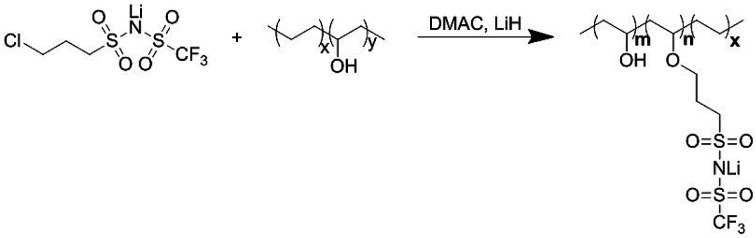

## 1. Introduction

Lithium ion batteries are considered as one of the most promising energy storage devices for consumer electronics and electric vehicles. The electrolyte plays a vital role in lithium ion batteries, providing channels for ionic charge carriers between the cathode and anode.^1^ To date, the conventional liquid electrolytes used in lithium ion batteries are prepared by dissolving LiPF_6_ in organic solvent,^2,3^ leading to the high ionic conductivity of around 10^−3^ S cm^−1^ at room temperature. However, severe safety issues arising from the flammability and potential leakage of the organic solvents have hampered the large scale utilization of lithium ion battery packs.^4^ To circumvent these issues, polymer electrolytes consisting of a high molecular weight polymer matrix blending with lithium salts have been proposed. Generally, polymer electrolytes possess a variety of advantages, such as low flammability, good thermal stability, improved safety and excellent electrochemical properties.^5,6^ However, similar to the conventional liquid electrolytes, both lithium ions and anions in such polymer electrolytes can migrate between the cathode and anode during the charge/discharge process.^7^ Besides, the interaction between the lithium cations with the heteroatoms such as N or O in the polymer matrix further lower the ion transference number of dual-ion polymer electrolytes.^8,9^ It causes a serious concentration polarization at the electrolyte/electrodes interface, restricting the high performance of lithium ion batteries especially at high current density.^10,11^

To circumvent these problems, single ion conductors with high lithium ion transference number (*t*_+_ ≈ 1) have been proposed to be utilized in lithium ion batteries, due to the intrinsic ability to significantly reduce the concentration polarization and restrict the growth of lithium dendrites during cycle performance.^12–14^ As for single ion conductors, the anions are covalently tethered onto polymer or inorganic backbones, which restricts the mobility of anions to maximize the transference number of lithium ions.^9,15^ Among them, ceramic electrolyte with high ionic conductivity at room temperature have captured much attention.^16–18^ However, the large interfacial resistance arising from the poor interfacial compatibility between the rigid ceramic and electrode materials cannot be completely solved in a short time,^19–22^ which makes it difficult to be applied in lithium ion batteries on a large scale. On the contrary, solid-state SIPE possesses the advantages of lighter weight and higher flexibility compared to the ceramic electrolytes, which is regarded as a better candidate.^12^ Unfortunately, the ionic conductivity of solid-state SIPEs is usually lower than the dual-ion polymer electrolytes, because the mobility of the lithium ion in the polymer matrix is difficultly at room temperature.^23^

An effective strategy to achieve high ionic conductivity is to saturate the SIPEs with appropriate organic solvents as plasticizers to facilitate lithium ion solvation and transport.^24–27^ In this regard, gel SIPE successfully combines the advantages of solid-state and liquid electrolytes, resulting in high ionic conductivity and excellent compatibility with electrodes.^28,29^ In the past several years, a variety of gel SIPEs based on sp^3^ hybrid boron,^28,29^ bis sulfonyl imide^30,31^ and sulfonate^32,33^ has been reported. For instance, Porcarelli *et al.*^24^ presented a simple method to prepare a cross-link gel SIPE *via in situ* radical copolymerization and the ionic conductivity was significantly enhanced compared to the dry state. Cui *et al.*^34^ developed a couple of sp^3^ hybrid boron based gel SIPEs and the assembled cell performed well at room temperature. These works have shown that gel SIPEs have great application potential in lithium ion batteries. However, most of the batteries utilizing gel SIPEs display poor cycling battery performance.^24,35–37^ Therefore, it is urgent and meaningful to develop novel gel SIPE by exploring other effective functional groups or polymer backbones.

**Scheme 1:**
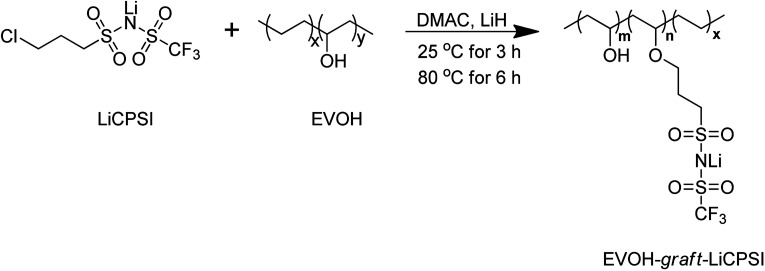
The synthetic route of EVOH-*graft*-LiCPSI.

In this work, a functional group of LiCPSI was synthesized and then grafted covalently on EVOH to immobilize the anions *via* Williamson's reaction as illustrated in Scheme 1. The LiCPSI was designed without unsaturated C=C bonds to enhance the electrochemical stability.^38^ Meanwhile, the flexible EVOH served as main chain to enable the excellent thermal and chemical stability. Thus, a side-chain grafted single ion polymer conductor (EVOH-*graft*-LiCPSI) was obtained. The SIPE blend film was prepared through blending the EVOH-*graft*-LiCPSI with the PVDF-HFP *via* solution cast method. The morphologies, thermal stability, mechanical strength and electrochemical properties of the gel SIPE have been investigated systematically.

## 2. Experimental section

### 2.1 Materials

Trifluoromethanesulfonamide and 3-chloropropanesulfonyl chloride were purchased from Aldrich and used without further purification. Acetonitrile, purchased from Aladdin, was dried with P_2_O_5_ and distilled under argon atmosphere at reduced pressure. Anhydrous lithium hydroxide (LiOH) and lithium hydride (LiH) were purchased from Aladdin. Dichloromethane and *N*-methyl-2-pyrrolidone (NMP, AR) were purchased from Sinopharm Chemical Reagent Co., Ltd. Mixed organic solvent of EC and DMC (v/v, 1/1) was purchased from Dodo Chem Co., Ltd. Poly(ethylene-*co*-vinyl alcohol) (EVOH) was purchased from Japan STS (average *M*_w_ ∼ 300 000, ethylene 35 wt%) and dried under vacuum at 80 °C for 12 h before use. Poly(vinylidene fluoride-*co*-hexafluoropropylene) (PVDF-HFP) was purchased from Sigma Aldrich Co., Ltd. (average *M*_w_ ∼ 400 000, average *M*_n_ ∼ 130 000). LiFePO_4_ (LFP) was obtained from Tianjin STL Energy Technology Co. Ltd.

### 2.2 Synthesis of lithium 3-chloropropanesulfonyl(trifluoromethanesulfonyl)imide (LiCPSI)

LiCPSI was synthesized according to the previous work.^39^ 2.034 g anhydrous lithium hydroxide and 6.314 g trifluoromethanesulfonamide were transferred into a 100 mL two-necked flask and placed in ice bath. 45 mL of dry acetonitrile as the solvent was added and the solution was stirred under argon protection. Then, 7.5 g 3-chloropropanesulfonyl chloride was slowly added *via* a dropping funnel. Afterwards, the reaction mixture was raised slowly to room temperature and left to react for 24 hours. The mixture was filtered and the filtrate was concentrated under vacuum to give faintly yellow solid. The product was further purified by recrystallizing in dichloromethane to obtain a pure white solid with an over ∼72% yield.

### 2.3 Synthesis of single ion polymer conductor (EVOH-*graft*-LiCPSI)

As shown in Scheme 1, 1 g of EVOH was dissolved in 20 mL of anhydrous DMAC with magnetic stirring at 25 °C to form a transparent solution. Then, 0.63 g of LiH was added under argon atmosphere. The reaction mixture was stirring for 3 hours followed by adding 2.2 g of LiCPSI, and further heated up to 80 °C for 6 hours. The solution was concentrated on a rotary evaporator and purified by dialysis against water for 3 days. Upon solvent removal, the EVOH-*graft*-LiCPSI was dried at 80 °C for 24 hours.

### 2.4 Characterization

1-Dimensional proton Nuclear Magnetic Resonance (^1^H NMR) spectroscopy of the compound was conducted on a 400 MHz NMR instrument (AVANCE III HD 400 MHz, Swiss BRUKER) with d_6_-DMSO as solvent at room temperature. Fourier Transform Infrared (FTIR) spectroscopy of the sample was investigated by a spectrophotometer (VERTEX 70 FTIR, Germany BRUKER) with the range from 4000 cm^−1^ to 750 cm^−1^ at room temperature. Thermogravimetric analysis of the electrolyte was evaluated by STA 409 PC (Germany NETZSCH) from ambient temperature to 600 °C with the heating rate of 10 °C min^−1^ under nitrogen atmosphere. The surface morphology of the blend membrane was probed by the scanning electron microscopy (SEM, SU8010, HITACHI). The prepared SIPE membrane was coated with gold in vacuum for 120 s prior to observation. Tensile strength of the blend film was measured with an electronic tensile tester (XLW (PC), Labthink, China) at room temperature.

### 2.5 Preparation of the SIPE membrane

The SIPE membrane was prepared *via* a solution casting method.^40^ Firstly, 0.1 g EVOH-*graft*-LiCPSI and 0.1 PVDF-HFP were added into 2 mL NMP to obtain a homogeneous solution after stirring for 24 hours at 60 °C. Then, the solution was subsequently cast onto a glass plate with an area of 20 cm^2^ followed by the solvent evaporation at 60 °C for several hours. After that, the SIPE membrane was peeled off from the glass plate and punched into small circles with a diameter of 19 mm. The SIPE membrane was further dried at 60 °C for 48 hours under vacuum. The gel SIPE was made by soaking certain amount of mixture solvent of EC/DMC (v/v, 1 : 1) without any additives in an argon filled glove box.

### 2.6 Solvent uptake of the SIPE membrane

Solvent uptake of the SIPE was measured by an improved weight method.^41^ The as-prepared SIPE was immersed in organic solvent of EC and DMC (v/v, 1/1) and weighted in the interval of 2 hours. The solvent uptake of the SIPE was calculated according to the eqn (1):1
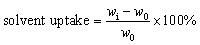
where *w*_i_ and *w*_0_ stand for the weights of the wet and the dry SIPEs, respectively. Mean solvent uptake was obtained as the average of three samples to reduce errors.

### 2.7 Electrochemical characterizations

#### 2.7.1 Ionic conductivity

Ion conductivity of the gel SIPE was measured *via* Electrochemical Impedance Spectroscopy (EIS) on a VMP3 electrochemical workstation by applying a 5 mV perturbation from 1 MHz to 100 mHz at the open circuit potential. The as-prepared gel SIPE was sandwiched between two stainless steel to assemble a symmetrical “SS|SIPE|SS” cell. The measurement was carried out between 25 and 80 °C with an interval of 10 °C. Ionic conductivity was calculated using eqn (2):2
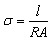
where *l* stands for the thickness of SIPE measured by a screw micrometer, *A* represents the area of the SIPE, *R* belongs to the bulk resistance, which was obtained from Nyquist plot and fitted with Z-View software.

#### 2.7.2 Ion transference number

Lithium-ion transference number was investigated *via* the symmetrical “Li|SIPE|Li” cell on a VMP3 electrochemical workstation with a static potential polarization of 10 mV. The value of lithium ion transference number (*t*_+_) was calculated from the combination of complex impedance and potentiostatic polarization methods as proposed by Evans *et al.*^42^ using eqn (3):3
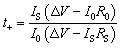
where Δ*V* is the potential applied across the cell, *I*_0_ and *I*_S_ are the initial and steady-state currents, and *R*_0_ and *R*_S_ represent the initial resistance and the steady-state resistance of the passivation layers on the lithium electrode, respectively.

#### 2.7.3 Electrochemical window

Electrochemical window of the SIPE was recorded in a “Li|SIPE|SS” cell on a VMP3 electrochemical workstation. Linear sweep voltammetry was conducted from 0 V to 6.0 V (*vs.* Li/Li^+^) at a constant rate of 1 mV s^−1^.

### 2.8 Battery test

The LiFePO_4_ composite cathode was prepared *via* a conventional casting method.^43^ 0.08 g of EVOH-*graft*-LiCPSI was dissolved in 1 mL of NMP. Then, 0.24 g of LiFePO_4_ and 0.08 g of acetylene black were added. The single ion conductor used as binder is to construct ion transport channel and reduce the interfacial resistance between the cathode and gel SIPE.^43^ The mixture was stirred 12 hours at room temperature to obtain homogeneous slurry, and then coated on an aluminum foil with a doctor blade. After drying in the oven, the cathode film was cut into circles with a diameter of 15 mm and further dried at 80 °C under vacuum for 12 hours. The active materials loading of the cathode film is around 0.8 mg cm^−2^. The 2025 coin-type cells were assembled in an argon filled glove box. After assembling, the cell was kept at 60 °C for 6 hours to ensure completely infiltration of carbonate solvents and well contact at the interfaces. The battery performances were evaluated by Land at various C-rates within the discharge and charge cut-off voltages of 2.5 V and 4.2 V at 25 °C.

## 3. Results and discussion

### 3.1 Characterizations

**Fig. 1:**
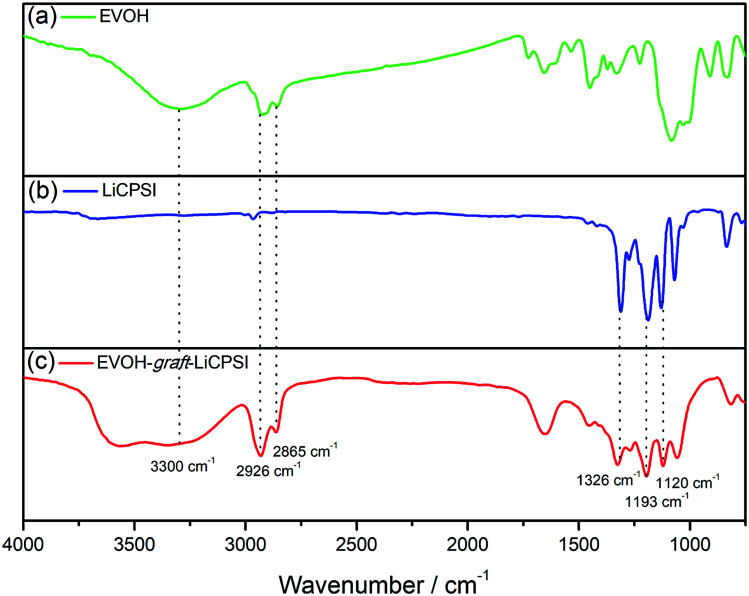
The FT-IR spectra. (a) EVOH, (b) LiCPSI and (c) EVOH-*graft*-LiCPSI.

Fig. 1a represents the typical FTIR spectra of EVOH. The peak at 3300 cm^−1^ is assigned to the stretch vibration of O–H groups. The peaks at 2865 cm^−1^ and 2926 cm^−1^ are ascribed to the antisymmetric and symmetric C–H stretching vibration of the CH_2_ groups. For LiCPSI (Fig. 1b), the peaks at 1326 cm^−1^, 1193 cm^−1^ and 1120 cm^−1^ are attributed to the asymmetric and symmetrical stretching of –SO_2_–, stretching of –S–N– and stretching of –S–N–S–, respectively. As expected, the infrared characteristic peaks of the bis sulfonyl imide functional group at around 1326 cm^−1^, 1193 cm^−1^ and 1120 cm^−1^ appear upon grafting (Fig. 1c). The results indicate that both LiCPSI and EVOH-*graft*-LiCPSI were successfully synthesized. However, the presence of O–H stretching vibration at 3300 cm^−1^ suggests the grafted degree is not 100%.

**Fig. 2:**
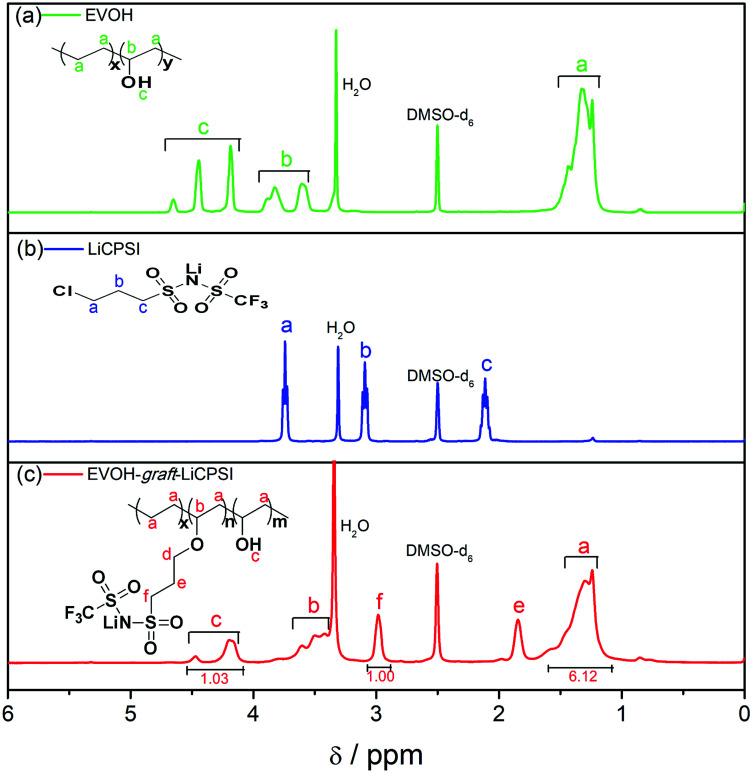
^1^H NMR spectra (a) EVOH, (b) LiCPSI and (c) EVOH-*graft*-LiCPSI.

To further examine the chemical structure of LiCPSI and EVOH-*graft*-LiCPSI, ^1^H NMR analysis was conducted (Fig. 2). As for pristine EVOH (Fig. 2a), peak ‘a’ (1.2–1.5 ppm) is assigned to the methylene proton. Peak ‘b’ (3.6–3.9 ppm) represents the methine proton. Peak ‘c’ (4.0–4.5 ppm) is ascribed to protons of hydroxyl. As for LiCPSI (Fig. 2b), peak ‘a’ (3.74 ppm), ‘b’ (3.10 ppm) and ‘c’ (2.15 ppm) represent the three types of methylene proton of Cl–CH_2_, CH_2_–SO_2_ and –CH_2_–, respectively. As for EVOH-*graft*-LiCPSI (Fig. 2c), peak ‘a’, ‘b’ and ‘c’ stand for the chemical shift of proton on EVOH. The appearance of peak ‘e’ (2.95 ppm) and ‘f’ (1.84 ppm) further demonstrates LiCPSI was successfully grafted on EVOH. Peak ‘d’ is not observed in Fig. 2a, which is probably covered by the water peak at 3.4 ppm. The grafted degree is calculated to be 33.3% based on the integrated area ratio of peak ‘c’ to peak ‘f’ *ca.* 1 : 1. Therefore, the lithium ion exchange capacity of EVOH-*graft*-LiCPSI is about 2.21 mmol g^−1^. To rigorously access the lithium ion concentration, the sample was measured by ICP-OES. The result is about 2.27 mmol g^−1^, slight higher than the theoretical value calculated according to the result of ^1^H NMR.

**Fig. 3:**
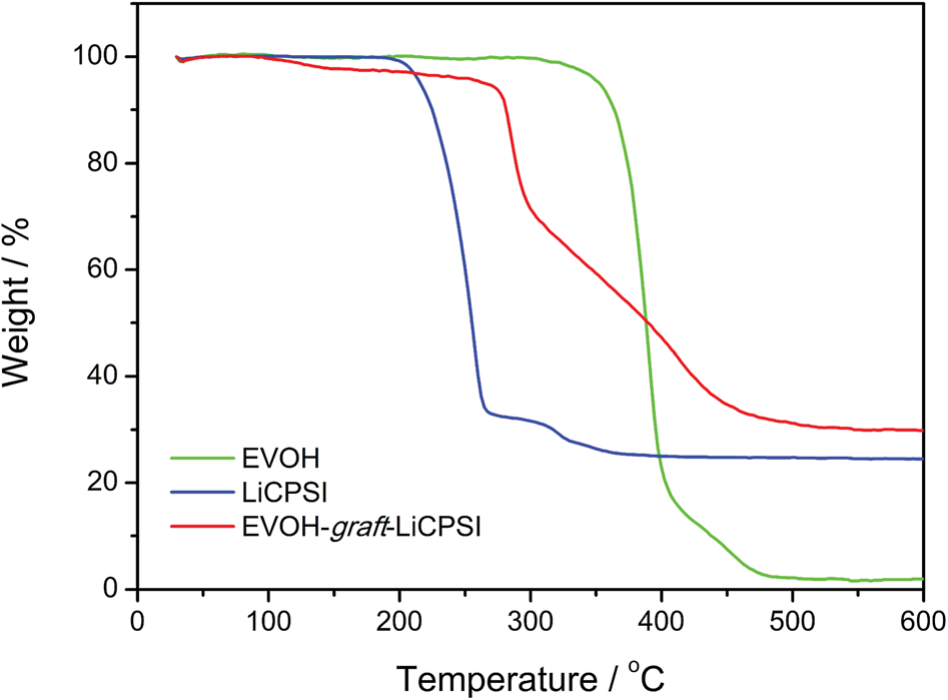
Thermo-gravimetry analysis of EVOH, LiCPSI and EVOH-*graft*-LiCPSI.

The thermal stability of EVOH, LiCPSI and EVOH-*graft*-LiCPSI are shown in Fig. 3. As seen from the TG curve (green curve), the onset mass loss temperature for EVOH is found to be 308 °C followed by a quick mass loss from 352 °C to 410 °C. The LiCPSI functional group shows high thermal stability up to 210 °C (blue curve). As for the EVOH-*graft*-LiCPSI, the corresponding TG curve (red curve) shows a weight loss of 5% at around 260 °C and then undergoes rapid weight loss till 450 °C. Obviously, the thermal stability of EVOH-*graft*-LiCPSI satisfies the requirement of the practical application in lithium ion batteries.^44^

### 3.2 Morphology

**Fig. 4:**
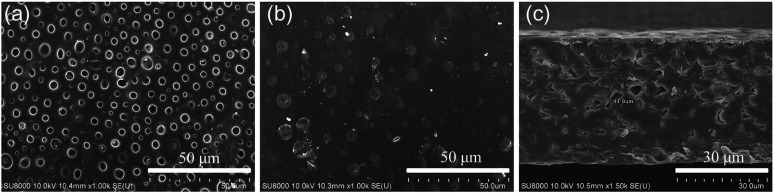
The SEM images of SIPE. (a) Front surface, (b) back surface and (c) cross section.

The micro morphologies of the dry SIPE were investigated by FE-SEM technology at room temperature as depicted in Fig. 4. It is found that the front surface of the SIPE exhibits a uniform porous structure (Fig. 4a), resulting from the solvent evaporation in the heating process. On the contrary, the back surface of the SIPE is dense in the micrometer scale (Fig. 4b). The cross section image of the SIPE exhibits a compact structure (Fig. 4c), indicating the good compatibility between EVOH-*graft*-LiCPSI and PVDF-HFP.^45^ In addition, the thickness of the SIPE is around 40 μm.

### 3.3 Tensile strength and solvent uptake capacity

**Fig. 5:**
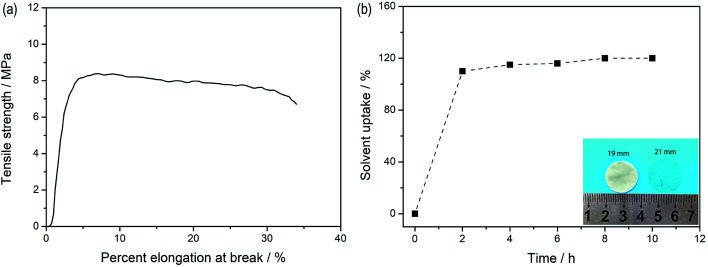
(a) Tensile test of the dry SIPE and (b) *ex situ* detection of solvent uptake for SIPE.

Fig. 5a depicts the stress–strain curves of the dry SIPE membrane. The tensile strength of the SIPE is found to be as high as 8.2 MPa with an elongation at break of 31%. The result indicates that the SIPE is sufficiently strong to withstand mechanical pressing upon the battery assembly and operation processes. When the dry SIPE was immersed into the organic solvent of EC/DMC (v/v, 1 : 1), the pale yellow membrane gradually become transparent (inset of Fig. 5b), indicating that the prepared SIPE is well compatible with the organic solvent.^45^ The corresponding *ex situ* detection of the solvent uptake of the SIPE is shown in Fig. 5b. The saturated solvent uptake of 122% is calculated after soaking for 8 h. It means that the mass of the solvent is about 55 percent of the total gel SIPE. Meanwhile, the diameter is enlarged from the original 19 mm to 21 mm after swelling (inset of Fig. 5b) and the surface extension ratio of the swollen film is 22%, probably arising from the stretch of the polymer chain, which is beneficial for the lithium ion transport.

### 3.4 Electrochemical performance

**Fig. 6:**
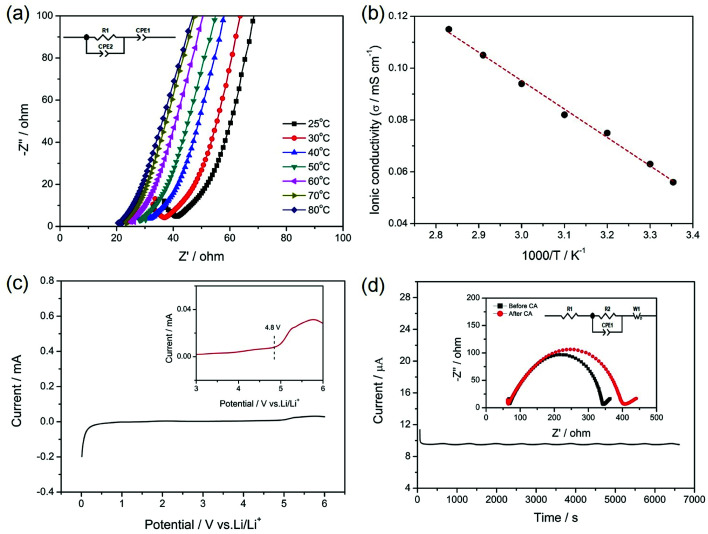
Electrochemical properties of the gel SIPE. (a) EIS plots of the gel SIPE; (b) ionic conductivity of the gel SIPE under different temperatures; (c) linear sweep voltammetry curve of the gel SIPE and (d) chronoamperometry (CA) of the gel SIPE with the EIS spectra recorded before and after CA test.

The ionic conductivity, electrochemical stability and lithium ion transference number of the gel SIPE are shown in Fig. 6. To investigate the influence of temperature on the ionic conductivity, the electrochemical impedance spectroscopy (EIS) was carried out from 25 °C to 80 °C. Fig. 6a displays the initial EIS spectra of the gel SIPE with the inset of the corresponding equivalent circuit. The intercept of a straight line at high frequency is attributed to the bulk resistance (*R*_1_), which is used for calculating the ionic conductivity of the gel SIPE. The ionic conductivities of the gel SIPE at different temperature are shown in Fig. 6b. The ionic conductivity of the gel SIPE is 5.7 × 10^−5^ mS cm^−1^ at 25 °C and increases to 1.1 × 10^−4^ mS cm^−1^ at 80 °C, showing a typical Arrhenius behavior.

**Fig. 7:**
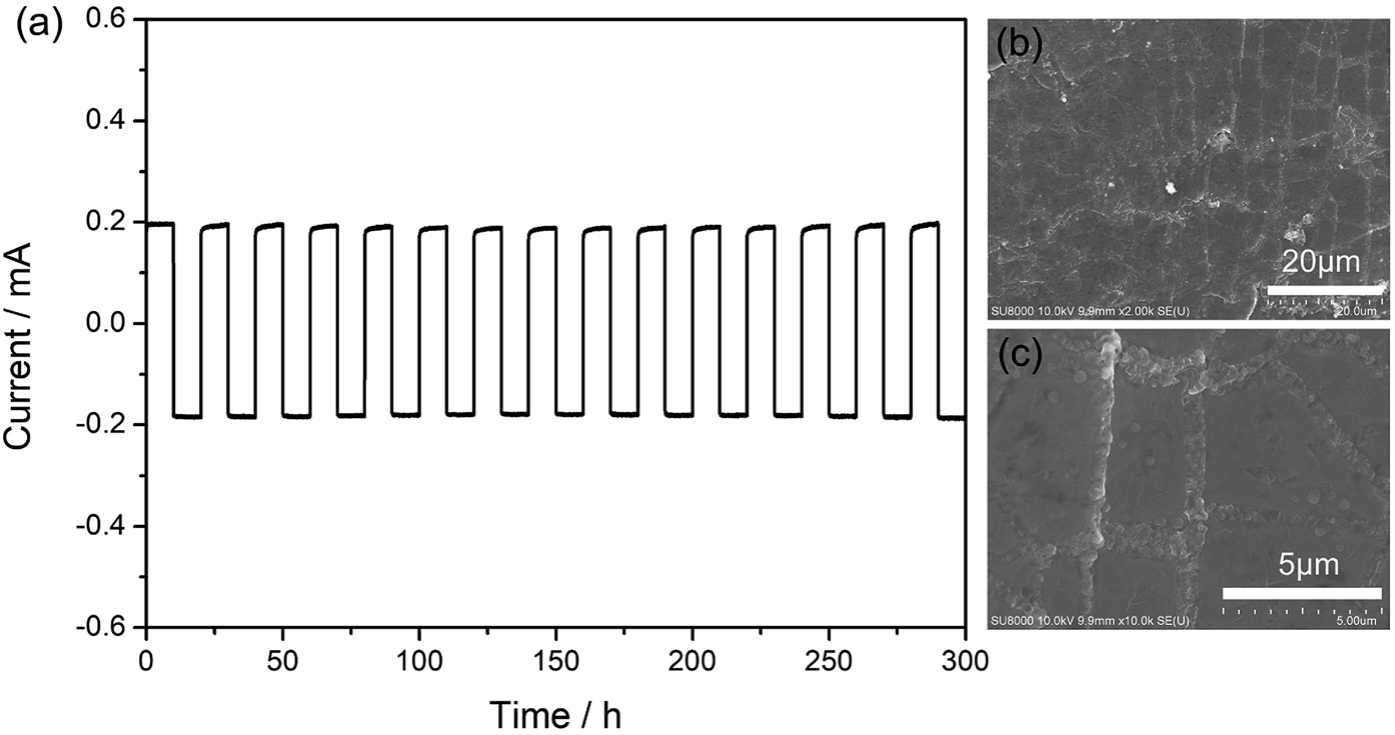
(a) Galvanostatic lithium plating/stripping cycling profiles of “Li|SIPE|Li” symmetric cell at density of 0.5 mA cm^−2^. (b) & (c) SEM image of lithium foil surface after lithium plating/stripping cycling for 300 hours.

The electrochemical window of gel SIPE is important for the choice of cathode materials. However, most of the reported single ion polymer conductors contains the rigid unsaturated C=C bonds, which may influence the electrochemical stability.^38,46^ Herein, to enhance the electrochemical stability of gel SIPE, a bis sulfonyl imide functional group without unsaturated C=C bonds was tethered to the flexible saturated polymer backbone of EVOH. As shown in Fig. 6c, the electrochemical stability window is stable up to 4.8 V (*vs.* Li/Li^+^) (Fig. 7a), which is well-suited for combination with LiFePO_4_ (ref. 47) and LiMn_2_O_4_ (ref. 47) cathode. The superior electrochemical stability is attributed to the absence of non-saturated bonds in the SIPE.^38^ However, the result is only slight higher than those works contains the rigid unsaturated C=C bonds,^41,45^ which is attributing to the existence of –OH groups in the polymer main chain.

Lithium ion transference number was determined by a dc polarization combined with impedance spectroscopy using a symmetrical “Li|SIPE|Li” cell. The measurement result is shown in Fig. 7b and the simulated equivalent circuit is also depicted in the inset. Clearly, the current response to the applied static potential polarization is recorded to be more than 7000 seconds. The initial current is 11.21 μA and stabilizes at the value of 9.65 μA after 200 s. Meanwhile, the corresponding interfacial resistance changes from 272 Ω to 335 Ω. Based on the results, the lithium ion transference number was calculated to be 0.88, indicating a typical single-ion conducting behavior.^45^ The high lithium transference number is owing to the large molecular weight EVOH as polymer backbones, which greatly restricts the movement of anions.

### 3.5 Galvanostatic lithium plating/stripping test

Galvanostatic cycling test was used to investigate the interface stability between the metal lithium foil and the gel SIPE in a symmetrical “Li|SIPE|Li” cell with the current density of 0.5 mA cm^−2^ at room temperature. As shown in Fig. 7a, the polarization potential of the symmetrical cell is 19 mV and almost remains constant for 300 hours, suggesting a stable interface during the testing process. Afterwards, the symmetrical cell was disassembled and the lithium foil was washed by anhydrous DMC. The cycled lithium foil was further investigated by scanning electron microscopy. As illustrated in Fig. 7b and c, the lithium foil shows smooth and dense surface morphology after galvanostatic cycling test. The results suggest that the gel SIPE is stable to metal lithium foil in the test period.

### 3.6 Battery performance

**Fig. 8:**
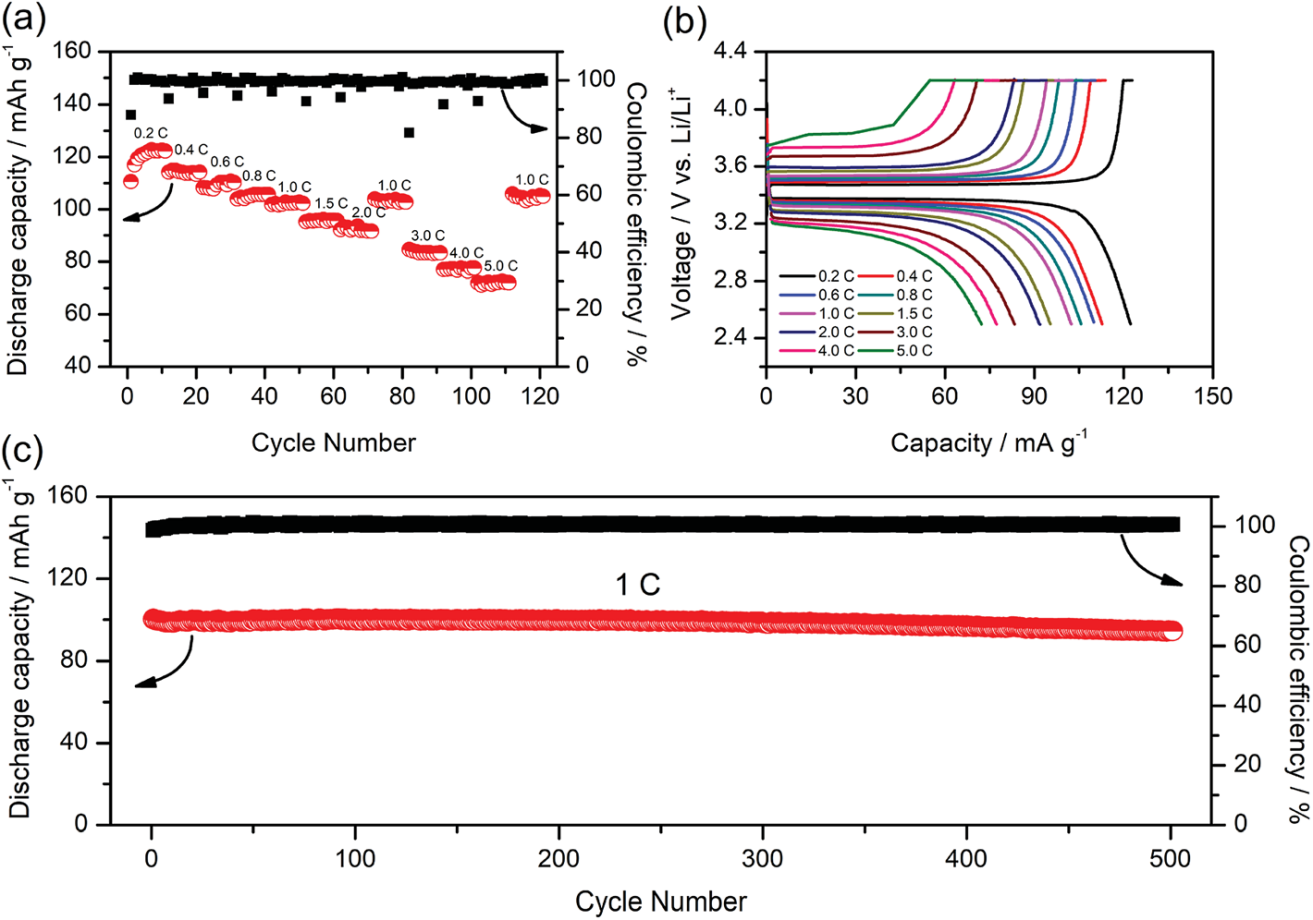
Battery performances of LiFePO_4_/Li cell at room temperature. (a) Rate performance at various C rates; (b) charge/discharge curves at different C rate; (c) cycling performances at 1C for 500 cycles.

To further study the performance of the gel SIPE in the cell, a LiFePO_4_/Li half-cell using the gel SIPE as electrolyte as well as separator was assembled and tested at room temperature. Fig. 8a presents the battery performances at different discharge rates from 0.2C to 5C. It can be seen that the LiFePO_4_/Li cell delivers a discharge capacity of 123 mA h g^−1^ at 0.2C, 115 mA h g^−1^ at 0.4C, 110 mA h g^−1^ at 0.6C, 106 mA h g^−1^ at 0.8C, 101 mA h g^−1^ at 1C, 96 mA h g^−1^ at 1.5C, 92 mA h g^−1^ at 2C, respectively. When the C-rate turns back to 1C, the discharge capacity can recover to 102 mA h g^−1^. Even at higher current rates, it still exhibits high capacities of 84 mA h g^−1^ at 3C, 77 mA h g^−1^ at 4C, 72 mA h g^−1^ at 5C. The coulombic efficiency at each C rate is close to 100%. The corresponding voltage plateaus at each C-rate are shown in Fig. 8b. The long-term cycle performance at 1C is shown in Fig. 8c. Before test, the battery was initially activated at 0.1C for several cycles. The discharge capacity at 1C stabilizes at 100 mA h g^−1^ during the first 250 cycles. After 500 cycles, the discharge capacity still remains at 95 mA h g^−1^, suggesting a 95% retention of the initial discharge capacity. The above battery test results reflect the excellent electrochemical performance of the gel SIPE, which is promising for practical application in lithium ion batteries.

## 4. Conclusion

In this work, we successfully synthesized a novel single ion polymer conductor by grafting the LiCPSI on the EVOH *via* Williamson's reaction. The SIPE membrane was prepared through blending the EVOH-*graft*-LiCPSI with the commercial PVDF-HFP. Organic solvent of EC/DMC as plasticizer was added to facilitate lithium ion dissociation and transport, resulting in a high ionic conductivity of 5.7 × 10^−5^ S cm^−1^ at 25 °C. The gel SIPE exhibits great electrochemical stability (4.8 V *vs.* Li/Li^+^) as well as good mechanical and thermal properties. In addition, due to the high lithium ion transference number of 0.88, the gel SIPE can effectively suppress the lithium dendrites growth as confirmed by the galvanostatic lithium plating/stripping cycling test. More importantly, the LiFePO_4_ half-cell assembled with the gel SIPE as the electrolyte as well as the separator delivers 100 mA h g^−1^ at 1C and remains 95 mA h g^−1^ after 500 cycles with a high capacity retention of 95%. We believe the SIPE synthesized by side chain grafting of bis sulfonyl imide functional groups to the commercial flexible polymer matrix is a promising choice in the future.

## Conflicts of interest

There are no conflicts to be declared.

## Supplementary Material
